# Gating control of the cardiac sodium channel Nav1.5 by its β3-subunit involves distinct roles for a transmembrane glutamic acid and the extracellular domain

**DOI:** 10.1074/jbc.RA119.010283

**Published:** 2019-10-28

**Authors:** Samantha C. Salvage, Wandi Zhu, Zaki F. Habib, Soyon S. Hwang, Jennifer R. Irons, Christopher L. H. Huang, Jonathan R. Silva, Antony P. Jackson

**Affiliations:** ‡ Department of Biochemistry, University of Cambridge, Cambridge CB2 1QW, United Kingdom; § Department of Biomedical Engineering, Washington University, St. Louis, Missouri 63130-489; ¶ Department of Physiology, Development and Neuroscience, University of Cambridge, Cambridge CB2 3EG, United Kingdom

**Keywords:** sodium channel, electrophysiology, protein structure, fluorescence, cardiomyopathy, cardiovascular disease, voltage clamp fluorescence

## Abstract

The auxiliary β3-subunit is an important functional regulator of the cardiac sodium channel Nav1.5, and some β3 mutations predispose individuals to cardiac arrhythmias. The β3-subunit uses its transmembrane α-helix and extracellular domain to bind to Nav1.5. Here, we investigated the role of an unusually located and highly conserved glutamic acid (Glu-176) within the β3 transmembrane region and its potential for functionally synergizing with the β3 extracellular domain (ECD). We substituted Glu-176 with lysine (E176K) in the WT β3-subunit and in a β3-subunit lacking the ECD. Patch-clamp experiments indicated that the E176K substitution does not affect the previously observed β3-dependent depolarizing shift of *V*_½_ of steady-state inactivation but does attenuate the accelerated recovery from inactivation conferred by the WT β3-subunit. Removal of the β3-ECD abrogated both the depolarizing shift of steady-state inactivation and the accelerated recovery, irrespective of the presence or absence of the Glu-176 residue. We found that steady-state inactivation and recovery from inactivation involve movements of the S4 helices within the DIII and DIV voltage sensors in response to membrane potential changes. Voltage-clamp fluorometry revealed that the E176K substitution alters DIII voltage sensor dynamics without affecting DIV. In contrast, removal of the ECD significantly altered the dynamics of both DIII and DIV. These results imply distinct roles for the β3-Glu-176 residue and the β3-ECD in regulating the conformational changes of the voltage sensors that determine channel inactivation and recovery from inactivation.

## Introduction

The voltage-gated sodium (Nav) channel is an intrinsic plasma membrane protein complex that initiates the action potential in electrically excitable cells. The Nav channel is composed of a 250-kDa α-subunit, which consists of four homologous domains (DI–DIV) containing the voltage-sensing domains (VSDs)^5^ and the ion-selective pore, in association with one or more auxiliary β-subunits that modify the gating behavior of the channel (1). In cardiac tissue, Nav1.5 is the predominant isoform. Mutations in Nav1.5 predispose to potentially fatal cardiac arrhythmias, such as the long QT and Brugada (BrS) syndromes (2). There is increasing evidence that the auxiliary β3-subunit is a major physiological regulator of the Nav1.5 α-subunit. For example, mice lacking β3 expression exhibit BrS-like cardiopathologies (3, 4). Furthermore, several BrS patients have been identified with mutations in the β3-subunit (5–8).

**Figure 1.**
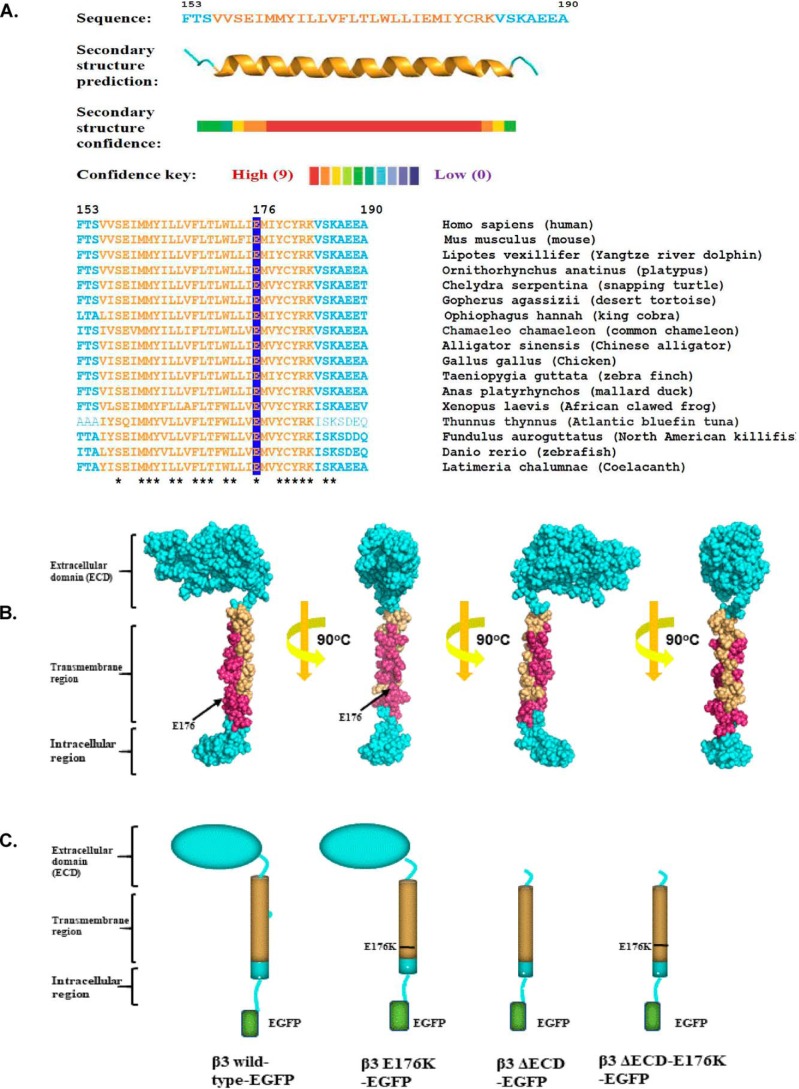
**Sequence analysis of β3-structures.** *A*, secondary structure prediction of the β3-subunit transmembrane region and its sequence alignment among a wide range of vertebrate species. Residue numbers refer to the human sequence. The predicted transmembrane region is colored *orange*, and the Glu-176 residue is *highlighted*. Residues fully conserved between species are indicated with *asterisks. B*, space-filling model of the β3-subunit with the fully conserved residues of the transmembrane region shown in *magenta*. Note that the helix face containing the Glu-176 residue is fully conserved. Analysis and modeling were as described under “Experimental procedures.” *C*, cartoon summary of the WT and mutant β3-subunit constructs used in this work and referred to in the text.

The β3-subunit contains an N-terminal extracellular domain (ECD), consisting of an Ig domain connected to a short, flexible, and probably disordered neck. This region is connected to a single transmembrane α-helix and a predominantly disordered intracellular C-terminal region (9, 10) (see Fig. 1). Recent structural evidence suggests that the DIII and DIV VSDs are implicated in the control of Nav channel inactivation (11, 12). Voltage-clamp fluorometry (VCF) experiments strongly imply that in Nav1.5, the β3-subunit influences the gating behavior of both these VSDs (13, 14). Consistent with this evidence, the β3-subunit accelerates Nav1.5 recovery from the inactivated state and produces a shift in the voltage, *V*_½_, of steady-state inactivation (13, 15). Although reports vary with respect to the direction of the voltage shifts, depending on the cell expression system used, they remain consistent with the β3-subunit altering the electrical field seen by one or more of the channel VSDs (16).

Mutagenesis and deletion experiments indicate that the β3-dependent shift in the *V*_½_ of inactivation requires the presence of the ECD (15). However, the role of the β3 transmembrane α-helix has been less well-studied. We have previously noted the presence of a highly conserved glutamic acid residue within the transmembrane region at position 176 (16). The location is intriguing because glutamic acids are rarely located in such hydrophobic regions (17). Here we mutate the Glu-176 residue to lysine in both the WT β3-subunit and the β3-subunit lacking the ECD. We examine the effects of these mutations on gating behavior and voltage-dependent movements of Nav1.5 DIII and DIV VSDs, using whole-cell patch-clamp electrophysiology and VCF. Our results suggest that the β3-subunit transmembrane α-helix lies close to the DIII VSD, where the Glu-176 residue plays an important role in facilitating recovery from inactivation. We further show that the β3-subunit extracellular region extends its influence both to the DIII and DIV VSDs. These data provide new structural and functional insights into how the β3-subunit can modulate both steady-state inactivation and recovery from inactivation.

## Results

### Structural insights into the Glu-176 residue and its location within the transmembrane domain

The amino acid residues referred to in the text are numbered from the start codon of the cDNA. Thus, the endoplasmic reticulum targeting signal is included in the numbering convention. Secondary structure predictions identified the likely position and extent of the transmembrane region as a conserved, hydrophobic, α-helical region extending from residues 156 to 180. The transmembrane region of single-pass, type 1 membrane proteins such as the β3-subunit are often enriched for residues such as tyrosine and cysteine on the membrane side of the cytoplasmic interface, followed on the cytoplasmic side by a cluster of basic residues (18). All these features are present in the β3 transmembrane region at positions 179–183 (Fig. 1*A*). Hence, the β3 transmembrane α**-**helix is likely to emerge into the cytosol at or close to residues Arg-182 and Lys-183. If so, the Glu-176 residue will be located almost two α-helical turns, or 9–10 Å, inside the membrane. Interestingly, the Glu-176 residue—which is fully conserved between all known β3 sequences—lies on a face of the α-helix that is also fully conserved (Fig. 1, *A* and *B*). This suggests the presence of an extended binding site along the transmembrane α-helix, in which the Glu-176 residue is likely to play an important role.

To investigate the function(s) of the Glu-176 residue, we used site-directed mutagenesis to replace it with lysine in the full-length β3 sequence. The lysine side chain has similar dimensions to the glutamic acid side chain but in aqueous media carries a positive charge. In a hydrophobic environment, the p*K_a_* of a lysine side chain is between 6 and 6.5 (19, 20). Hence, even if buried within the membrane, a significant minority of the lysine should remain positively charged and thus likely to be electrostatically distinct from the WT glutamic acid. To investigate the potential for functional synergy between the Glu-176 residue and the extracellular domain, we also introduced the E176K mutant into a β3 deletion mutant lacking the extracellular domain (15). All β3 constructs contained a C-terminal EGFP tag to aid the identification of transfected cells. A diagrammatic summary of the mutational constructs used in this work and their naming convention is shown in Fig. 1*C*.

### The Glu-176 residue is not required for β3-subunit self-oligomerization

**Figure 2.**
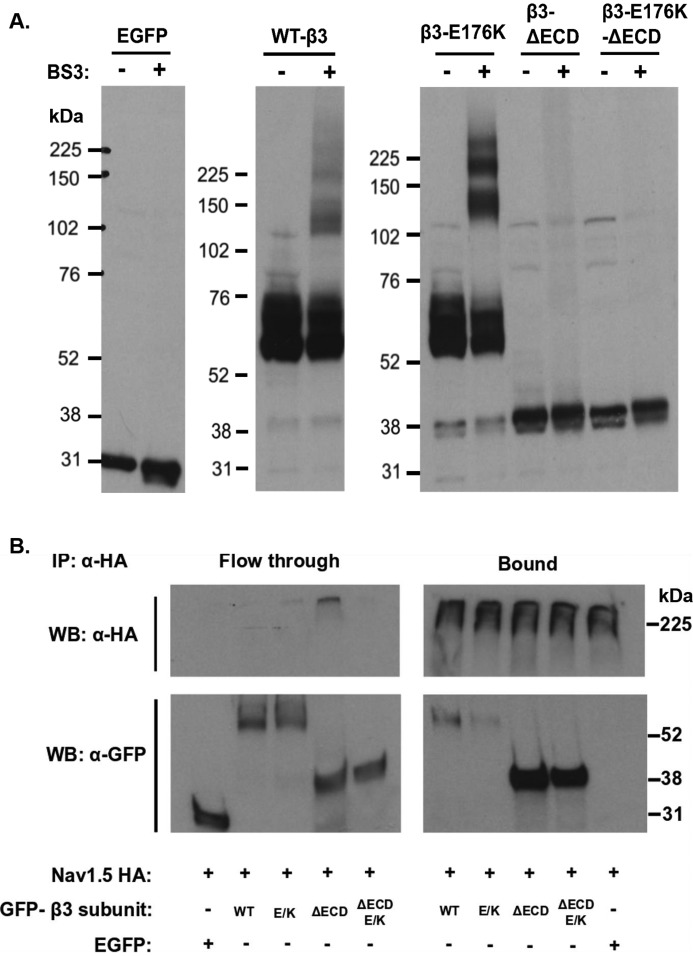
**β3 homooligomerization and Nav1.5α–β3 interaction.** *A*, Western blots (*WB*) of cell lysates singly transfected with WT-β3-EGFP or mutant subunits, cross-linked with BS3. The monomeric full-length β-subunits tagged with EGFP were run at ∼58–75 kDa. The multiple bands are indicative of variations in glycosylation patterns. With BS3, dimeric and trimeric forms appear at ∼120–130 and 180–200 kDa, respectively. *B*, co-IP of cell lysates co-transfected with Nav1.5 HA- and EGFP-tagged β3-subunits or control EGFP alone. The samples were immunoprecipitated (*IP*) with a monoclonal anti-HA. (Nav1.5) antibody coupled to protein G–agarose beads. The bound and flow-through (supernatant) fractions were separated on SDS-PAGE and blotted for HA and EGFP. β3-EGFP with and without the ECD and E176K mutation were pulled down with the Nav1.5 HA. The EGFP is only present in the unbound, flow-through fraction.

The isolated β3-subunit Ig domain can form trimers in solution and the full-length β3-subunit forms trimers in the plasma membrane when expressed in HEK293 cells in the absence of the Nav channel α-subunit (21). We have previously suggested that the β3-subunit trimer could be stabilized by hydrogen bonding between the membrane-embedded and protonated Glu-176 side chains on adjacent transmembrane domains (16). To test this hypothesis, cross-linking experiments with the membrane-impermeant bis-sulfosuccinimidyl suberate (BS3) were carried out on HEK293F cells singly transfected with the full-length WT β3-subunit and the different β3-subunit mutations (Fig. 2*A*).

In the absence of the cross-linker BS3, WT-β3-EGFP and E176K-EGFP migrated as monomers on SDS-PAGE (Fig. 2*A*). The presence of closely spaced bands is due to multiple glycosylation states (22). Similarly, under these conditions the β3-ΔECD-EGFP and the β3-ΔECD-E176K-EGFP proteins were observed as monomers at ∼35–38 kDa, In the presence of BS3, extra bands were observed for the WT-β3-EGFP and E176K-EGFP β3-subunits at ∼130–140 and 180–200 kDa, indicating the formation of dimers and trimers. There was no obvious reduction in the extent of cross-linking between WT and E176K mutant, suggesting that the mutation does not prevent the ability to form dimers and trimers. However, oligomerization induced by cross-linking was lost with the removal of the ECD, whether or not the resulting ΔECD construct contained the E176K mutation in its transmembrane region. Hence, oligomerization depends more on the presence of an intact ECD than on the transmembrane region. We found no evidence that the homophilic interactions between individual β3-subunits specifically required the presence of a glutamic acid at position 176.

### Binding of the β3-subunit to the Nav1.5 α-subunit persists with the E176K mutation

Binding of the different β3-subunit variants to the Nav1.5 α-subunit was examined by co-immunoprecipitation experiments. Here, a Nav1.5 α-subunit with a C-terminal HA tag (21) was co-transfected with the WT or mutant β3-subunits. This resulted in the co-immunoprecipitation of all co-expressed β3-subunits, including the β3-ΔECD-E176K-EGFP mutant that both lacked the extracellular Ig domain and contained the E176K change (Fig. 2*B*). This suggests that neither the extracellular Ig domain nor the Glu-176 residue alone are essential for the binding of the β3-subunit to the Nav1.5 α-subunit. Interestingly, it was noticeable that the intensity of the immunoprecipitated β3-E176K EGFP mutant in the bound fraction was somewhat weaker compared with WT-β3 EGFP, whereas the immunoprecipitated β3-ΔECD (with or without the E176K mutant) was noticeably more intense. Hence, the Glu-176 residue and the ECD may influence the overall stability of the Nav1.5–β3 complex with Nav1.5 in different ways (see “Discussion”).

### Loss of the ECD, but not of the Glu-176 residue, abolishes the effect of the β3-subunit on the voltage sensitivity of Nav1.5 inactivation gating

**Figure 3.**
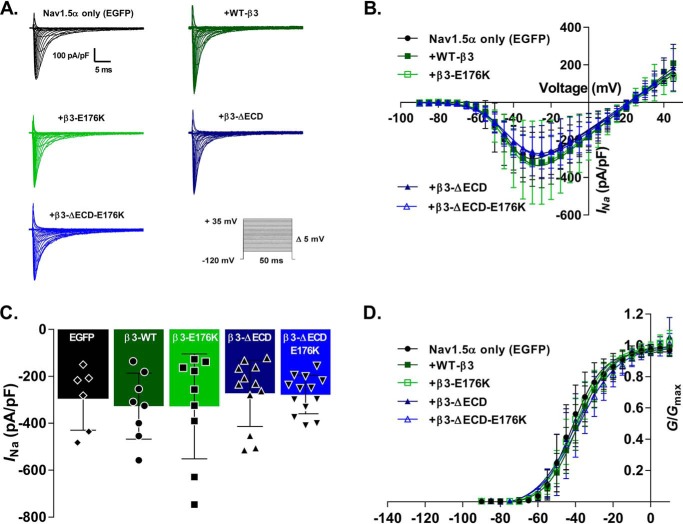
**Nav1.5 steady-state activation properties with and without WT or mutant β3.** *A*, representative whole-cell sodium current recordings from HEK293F-Nav1.5 cells transfected with WT and mutant β3-subunits in response to a steady-state activation protocol (*inset*). *B*, *I*/*V* Boltzmann curves (described under “Experimental procedures”) of Nav1.5 currents normalized to cell capacitance. *C*, histograms showing the Nav1.5 peak current densities (*I*_Na_) with and without WT and mutant β3-subunits. *D*, channel conductance as a function of voltage. *Curves* are fit to a Boltzmann function; *V*_½_ and *k* were both unaffected by the presence of the β3-subunit or any of the mutants. For *B–D*, the data are means ± S.D. (*n* ≥ 6; see Table 1 for individual groups), and statistical significance was tested with one-way ANOVA. All parameters (peak *I*_Na_, *V*_½_, and *k*) showed no statistically significant variation (*p* > 0.2). See Table 1 for individual values.

Stable Nav1.5-HEK293F cells were co-transfected with an empty EGFP vector (Nav1.5 with EGFP) or EGFP-tagged WT or mutant β3-subunits (*i.e.* Nav1.5 with WT-β3-EGFP; Nav1.5 with β3-E176K-EGFP; Nav1.5 with β3-ΔECD-EGFP; and Nav1.5 with β3-ΔECD-E176K-EGFP). Representative traces of whole-cell sodium currents (*I*_Na_) in response to an activation protocol are shown in Fig. 3*A*. None of the β3-subunit variants resulted in significant changes in peak *I*_Na_, voltage, *V*_½_ of maximal activation or slope factor, and *k* (*p* > 0.05; Fig. 3, *B–D*, and Table 1).

**Table 1:** **Nav1.5 activation and steady-state inactivation and recovery from inactivation parameters with and without the β3 WT and mutant subunits** Activation (*G*/*G*_max_) and inactivation data are fit to the Boltzmann function, and *V*_½_ and *k* were derived from this. Peak *I*_Na_ is the mean of the absolute maximum *I*_Na_ elicited by each cell during the activation protocol. All data are means ± S.D. (*n* ≥ 6, indicated in the table), compared using one-way ANOVA (*p* > 0.2 for all activation parameters, and *p* < 0.01 for inactivation parameters). Parameters that were determined to be statistically significant were subjected to a Sidak's multiple comparison post hoc test (all conditions were compared against Nav1.5 + EGFP and Nav1.5 + β3-EGFP).

HEK293F cells	Activation (*G*/*G*_max_)				Inactivation		
	Peak *I*_Na_	*V*_½_	*k*	*n*	*V*_½_	*k*	*n*
		*mV*	*mV*		*mV*	*mV*	
Nav1.5 + EGFP	−295.6 ± 134.2	−41.42 ± 6.29	7.31 ± 1.92	6	−82.21 ± 4.81*^a^*	−8.07 ± 0.86*^b^*	10
Nav1.5 + FL β3-EGFP	−327.0 ± 140.6	−38.63 ± 5.49	8.03 ± 1.93	8	−75.2 ± 4.00	−6.91 ± 0.75	13
Nav1.5 + FL β3-E176K-EGFP	−328.0 ± 223.9	−40.29 ± 4.55	8.2 ± 0.79	9	−76.28 ± 2.22*^c^*	−7.33 ± 0.67	10
Nav1.5 + ΔECD-β3-EGFP	−272.6 ± 141.2	−40.14 ± 6.48	9.09 ± 2.06	12	−80.06 ± 5.28*^b^*	−7.99 ± 0.90*^b^*	13
Nav1.5 + ΔECD-β3-E176K-EGFP	−278.1 ± 81.85	−38.01 ± 7.12	9.41 ± 2.59	13	−81.36 ± 4.64*^a^*	−7.79 ± 0.92	13

*^a^p* < 0.01 compared with Nav1.5 + β3.

*^b^ p* < 0.05 compared with Nav1.5 + β3.

*^c^ p* < 0.01 compared with Nav1.5.

**Figure 4.**
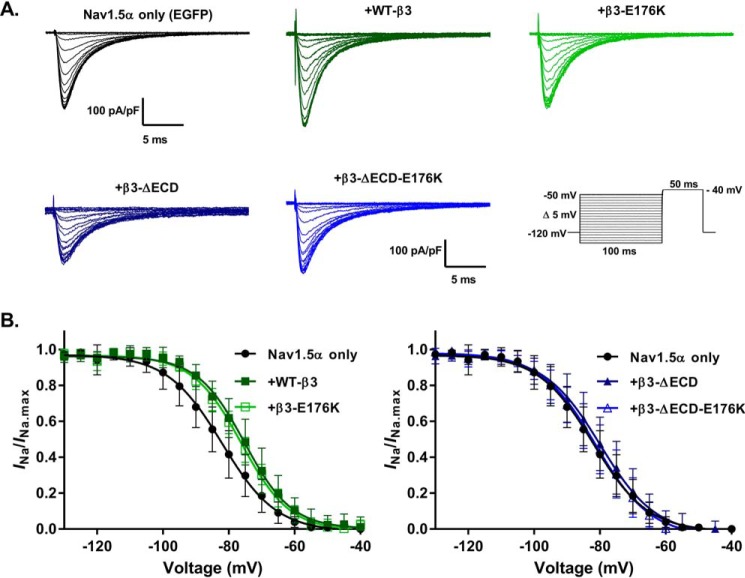
**Effects of the β3-subunit on Nav1.5 steady-state inactivation are mediated through the ECD.** *A*, representative whole-cell Na^+^ currents in response to a steady-state inactivation protocol (*inset*). *B*, peak *I*_Na_ from each sweep normalized to the maximum peak *I*_Na_ (*I*_Na_/*I*_Na.max_) of all sweeps and plotted as a function of the pre-pulse voltage step. The data are means ± S.D. (*n* ≥ 10, see Table 1 for individual values) and are separated by Nav1.5 + EGFP, WT-β3-EGFP, and β3-E176K-EGFP in the *left panel* and Nav1.5 + EGFP, ΔECD-β3-EGFP, and ΔECD-β3-E176K-EGFP in the *right panel*. The Nav1.5 + EGFP is the same in both graphs; the separation is for clarity. The *curves* are fit to Boltzmann functions (see “Experimental procedures”). The statistical significance of the *V*_½_ and *k* values produced were determined using one-way ANOVA (both *V*_½_ and *k*; *p* < 0.01) followed by a Sidak's multiple comparison post hoc test (all conditions were compared against Nav1.5 + EGFP and Nav1.5 + β3-EGFP). *V*_½_ of Nav1.5 steady-state inactivation is positively shifted by the full-length β3 and the β3 with the single transmembrane E176K point mutation (Nav1.5 + EGFP *versus* WT-β3, *p* = 0.0026; and Nav1.5 + EGFP *versus* β3-E176K-EGFP, *p* = 0.0263). Removal of the Ig-like ECD abolishes these shifts (Nav1.5 + EGFP *versus* β3-ΔECD-EGFP, *p* = 0.865; and Nav1.5 + EGFP *versus* β3-ΔECD-E176K-EGFP, *p* = 0.99). See Table 1 for all comparisons.

Typical inactivation traces are shown in Fig. 4, and the parameters are summarized in Table 1. Expression of WT-β3-EGFP resulted in a ∼7-mV depolarizing shift of Nav1.5 steady-state inactivation, agreeing with previous findings (13) expressing that β3-E176K-EGFP did not alter this effect. However, the loss of the extracellular Ig domain completely abolished this shift, independent of the presence of the Glu-176 residue. The slope factor, *k*, exhibited a small but significant decrease in the presence of WT-β3-EGFP, suggesting a slight increase in voltage dependence of inactivation.

### Both loss of the ECD and the E176K mutation abrogate the β3-mediated acceleration of recovery from Na^+^ current inactivation

**Figure 5.**
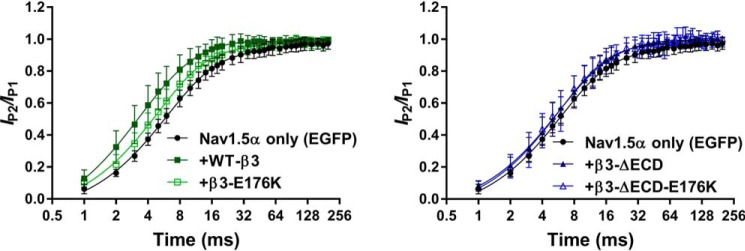
**Acceleration of Nav1.5 recovery from inactivation by β3 is abolished with loss of the ECD or the transmembrane glutamic acid.** Recovery from inactivation is expressed as the fraction of current produced by a second pulse over time following an identical pre-pulse (see “Experimental procedures”). The data are means ± S.D. (*n* ≥ 7, see Table 2) fit to double exponential functions, and the parameters (*k*, τ, and *t*_½_ for both the fast and slow components) are compared with one-way ANOVA (*p* ≤ 0.002 for all) followed by a Sidak's multiple comparison post hoc test (all conditions were compared against Nav1.5 + EGFP and Nav1.5 + β3-EGFP). Nav1.5 + EGFP, WT-β3-EGFP, and β3-E176K-EGFP are in the *left panel*, and Nav1.5 + EGFP, ΔECD-β3-EGFP, and β3-ΔECD-E176K-EGFP are in the *right panel*. The Nav1.5 + EGFP is the same in both graphs; the separation is for clarity. Nav1.5 recovery from inactivation was accelerated by the presence of the WT full-length β3-subunit (Nav1.5 + EGFP *versus* WT-β3-EGFP, *p* < 0.001 for all fast and slow components). This effect was lost with the full-length β3-E176K mutation (Nav1.5 + EGFP *versus* β3-E176K-EGFP, *p* > 0.05) and loss of the ECD (Nav1.5 + EGFP *versus* β3-ΔECD). See Table 2 for full details.

It has previously been shown that the β3-subunit accelerates Na^+^ channel recovery from inactivation (13, 23). We sought to determine whether either the extracellular Ig domain or the Glu-176 residue influenced this mechanism. Representative whole-cell Na^+^ currents were elicited by a double-pulse protocol, whose pulses were separated by progressively incremental time intervals, Δ*t*, are shown in Fig. 5. These respective P1 and P2 pulses yielded peak currents *I*_P1_ and *I*_P2_. The degrees of recovery following different time intervals, Δ*t*, were compared by obtaining the ratio of *I*_P2_/*I*_P1._ Plots of *I*_P2_/*I*_P1_ as a function of time are fitted to a double exponential, comprising a fast and slow component, as described under “Experimental procedures.” Both the fast and slow components of Nav1.5 recovery were accelerated by the co-expression of WT-β3-EGFP compared with those obtained with the co-expression of Nav1.5 with EGFP alone (*p* < 0.005; Fig. 5 and Table 2). The β3-E176K mutation significantly attenuated both of these effects but did not completely abolish it. The loss of the extracellular Ig domain also resulted in a slowing of recovery from inactivation compared with WT-β3-EGFP. That was more pronounced in the fast component. Hence, the kinetic and steady-state data together indicate overlapping but distinct roles for the Glu-176 residue and the extracellular Ig domain of β3 in regulating recovery from inactivation.

**Table 2:** **Nav1.5 recovery from inactivation parameters with and without the β3 WT and mutant subunits** Recovery from inactivation data is fit to a double exponential function. All data are means ± S.D. (*n* ≥ 7, indicated in the table). Statistically significant results were determined using one-way ANOVA (all parameters *p* ≥ 0.0006) and compared with a Sidak's multiple comparison post hoc test (all conditions were compared against Nav1.5 + EGFP and Nav1.5 + β3-EGFP).

HEK293F cells	Recovery from inactivation						
	*k*_fast_	*k*_slow_	τ_fast_	τ_slow_	*t*_½ fast_	*t*_½ slow_	*n*
			*ms*	*ms*	*ms*	*ms*	
Nav1.5 + EGFP	0.175 ± 0.025*^a^*	0.028 ± 0.015*^a^*	5.83 ± 0.848*^a^*	49.14 ± 31.506*^b^*	4.041 ± 0.588*^a^*	34.06 ± 21.837*^a^*	12
Nav1.5 + FL β3-EGFP	0.429 ± 0.181	0.134 ± 0.081	2.807 ± 1.32	12.04 ± 11.011	1.942 ± 0.910	8.34 ± 7.632	9
Nav1.5 + FL β3-E176K-EGFP	0.222 ± 0.064*^b^*	0.039 ± 0.028*^b^*	4.81 ± 1.262	40.12 ± 27.38	3.335 ± 0.875	27.81 ± 18.976	7
Nav1.5 + ΔECD-β3-EGFP	0.203 ± 0.063*^a^*	0.059 ± 0.033*^b^*	5.442 ± 2.011*^b^*	22.23 ± 11.917*^c^*	3.772 ± 1.394*^b^*	15.41 ± 8.26*^c^*	10
Nav1.5 + ΔECD-β3-E176K-EGFP	0.226 ± 0.112*^a^*	0.07 ± 0.054*^d^*	5.182 ± 1.870*^b^*	23.69 ± 15.821	3.592 ± 1.296*^b^*	16.42 ± 10.966	10

*^a^p* < 0.001 compared with Nav1.5 + β3.

*^b^ p* < 0.01 compared with Nav1.5 + β3.

*^c^ p* < 0.05 compared with Nav1.5.

*^d^ p* < 0.05 compared with Nav1.5 + β3.

### Distinct roles for the Glu-176 residue and the ECD in modulating DIII and DIV VSD movement

The Nav channel VSDs are comprised of the first four transmembrane α-helices (S1–S4) within each of the four internally homologous Nav channel domains (DI–DIV) (Fig. S1). Changes in membrane potential initiate movement of the charged S4 helix within each VSD. This enables channel opening (for the case of DI–DIII VSDs) and subsequent channel inactivation (for the case of DIV VSD) (1). The regulatory effect of the β3-subunit on recovery from inactivation arises at least in part by its modulation of the voltage responses of the DIII and DIV VSDs (13, 14). Further investigations into the effect of the Glu-176 residue upon these two VDSs utilized VCF in cut-open oocytes. Two separate constructs (M1296C and S1618C) were generated in the Nav1.5 α-subunit by site-directed mutagenesis. The M1296C introduced a cysteine into the extracellular loop connecting helices S3 and S4 of the DIII VSD; the S1618C mutation introduced a cysteine into the extracellular loop connecting helices S3 and S4 of the DIV VSD (13, 24) (Fig. S1). These cysteines were selectively labeled with the fluorophore MTS-TAMRA. Changes in fluorescence intensity (Δ*F*) of the TAMRA adjunct were used to track conformation changes of the S4 helix in the DIII and DIV VSDs in response to changes in membrane potential (13). Previous experiments established that cysteine introduction and labeling of the M1296C and S1618C Nav1.5 constructs caused only small changes in channel gating properties compared with WT Nav1.5 (24). Moreover, co-expression of the β3-subunit with MTS-TAMRA labeled M1296C and S1618C Nav1.5 constructs caused similar gating shifts as with the WT Nav1.5 channel (13). On this basis, we conclude that the interactions between Nav1.5 α-subunit and the β3-subunit are not fundamentally compromised by the VCF mutations or the MTS-TAMRA labeling.

**Figure 6.**
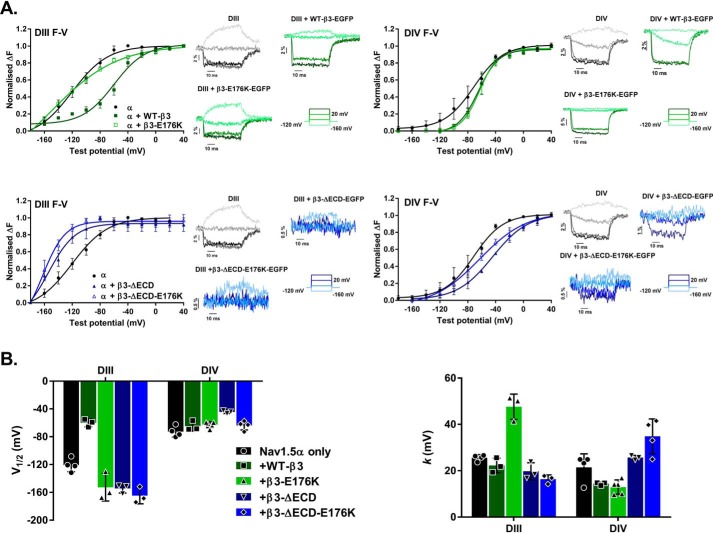
**Effects of the β3-subunits on Nav1.5 VSD movements as detected by VCF.** *A*, voltage dependence of fluorescence changes in DIII and DIV of Nav1.5. All *curves* are fit with a Boltzmann function, and the corresponding data points are means ± S.D. (*n* ≤ 3, see Table 3) compared by one-way ANOVA (all parameters *p* ≤ 0.0005) and followed by a Sidak's multiple comparison post hoc test (all conditions were compared against Nav1.5 + EGFP and Nav1.5 + β3-EGFP; see Table 3 for full results). *Upper left panel*, normalized change in DIII fluorescence plotted (means ± S.D.) as a function of test potential (*F–V* curve) for Nav1.5 α alone and with WT-β3 or β3-E176K. WT-β3 induces a depolarizing shift in the *F–V* curve compared with Nav1.5 alone (Nav1.5 *versus* Nav1.5 + β3-EGFP; *p* = 0.0002), an effect lost in the presence of the E176K mutation (Nav1.5 + β3-EGFP *versus* Nav1.5 + β3-E176K-EGFP; *p* < 0.0001). Adjacent *insets* show corresponding representative fluorescence signals and test protocol. *Upper right panel*, DIV *F–V* curve for α alone and with WT-β3 or β3-E176K. The presence of either WT-β3 or β3-E176K induces a depolarizing rightward shift in the initial activation of DIV, but the movement is accelerated compared with Nav1.5 alone. Adjacent *insets* show corresponding representative VCF traces and test protocol. *Lower left panel*, DIII *F–V* curve for α alone or with β3-ΔECD or β3-ΔECD-E176K. The loss of the ECD results in a hyperpolarizing leftward shift (Nav1.5 + β3-EGFP *versus* Nav1.5 + β3-ΔECD, *p* < 0.0001; and Nav1.5 + β3-EGFP *versus* Nav1.5 + β3-ΔECD-E176K-EGFP, *p* < 0.0001). Adjacent *insets* show the corresponding representative fluorescence signals, where the movement of the DIII sensor is noticeably attenuated by the presence of either β3-subunits lacking the ECD. *Lower right panel*, DIV *F–V* curve for α alone or with β3-ΔECD or β3-ΔECD-E176K. β3-ΔECD and β3-ΔECD-E176K co-expression result in differing degrees of rightward depolarizing shift of the curve. Adjacent *insets* show the corresponding representative fluorescence signals, where the movement of the DIV sensor like the DIII sensor is noticeably attenuated. *B*, histograms of the mean (± S.D., *n* ≥ 3) *V*_½_ (*left panel*) and *k* (*right panel*) values for DIII and DIV from the Boltzmann curve fits of the data in *A*. Table 3 has the full numerical values and statistical comparisons.

For the DIII VSD, the presence of the full-length, WT β3-subunit (WT-β3-EGFP) resulted in the activation of DIII VSD occurring at less negative potentials but at a similar rate. This produced a significantly depolarized *V*_½_ of the S4 helix movement, compared with Nav1.5 alone. This effect was abrogated by expression of the β3-E176K-EGFP, which activated the DIII VSD at similar potentials to Nav1.5 expressed alone, but at a slower rate. This suggests the Glu-176 residue is important in mediating the effect of the β3-subunit on DIII VSD modulation. In contrast, the β3-ΔECD-EGFP mutant and the β3-ΔECD-E176K-EGFP double mutant activated the DIII VSD at more hyperpolarized potentials; this was such a drastic effect that it produced a significantly hyperpolarized shift of *V*_½_ even compared with Nav1.5 expressed in the absence of the β3-subunit. However, there does not appear to be an additive effect in the double mutation (Fig. 6 and Table 3). The slope factor (*k*), which is inversely proportional to charge movement across the membrane (see “Discussion”), was almost doubled by the β3-E176K-EGFP mutant, yet the β3-ΔECD-EGFP and the β3-ΔECD-E176K-EGFP mutants, as well as the WT-β3-EGFP subunit, had little effect on this parameter compared with Nav1.5 expressed alone (Fig. 6 and Table 3).

**Table 3:** **VCF data for DIII and DIV in Nav1.5 with and without the β3 WT and mutant subunits** The *V*_½_ and *k* values for DIII and DIV are derived from the Boltzmann fit of the normalized change in fluorescence as a function of voltage. All data are means ± S.D. (*n* ≥ 3 indicated in the table). Statistically significant results were determined using one-way ANOVA (all parameters *p* ≤ 0.0005) followed by Sidak's multiple comparison post hoc test (all conditions were compared against Nav1.5 + EGFP and Nav1.5 + β3-EGFP).

Cut-open oocytes	DIII F-V			DIV F-V		
	*V*_½_	*k*	*n*	*V*_½_	*k*	*n*
	*mV*	*mV*		*mV*	*mV*	
Nav1.5	−120.68 ± 9.57*^a^*	25.6 ± 1.32	4	−72.77 ± 7.79	21.43 ± 5.87	4
Nav1.5 + FL β3-EGFP	−60.04 ± 5.68	22.26 ± 3.14	3	−64.87 ± 7.04	14.38 ± 0.65	3
Nav1.5 + FL β3-E176K-EGFP	−152.7 ± 19.71*^a^*^,^*^b^*	47.59 ± 5.47*^a^*^,^*^c^*	3	−62.66 ± 4.32	12.93 ± 3.08	5
Nav1.5 + ΔECD-β3-EGFP	−154.4 ± 6.51*^a^*^,^*^b^*	19.75 ± 3.68	3	−44.34 ± 2.17*^c^*^,^*^d^*	25.67 ± 0.96	3
Nav1.5 + ΔECD-β3-E176K-EGFP	−164.8 ± 11.67*^a^*^,^*^e^*	16.31 ± 1.81*^b^*	3	−63.68 ± 6.65	34.84 ± 7.51*^a^*^,^*^e^*	4

*^a^p* < 0.01 compared with Nav1.5 + β3.

*^b^ p* < 0.05 compared with Nav1.5.

*^c^ p* < 0.001 compared with Nav1.5.

*^d^ p* < 0.01 compared with Nav1.5 + β3.

*^e^ p* < 0.01 compared with Nav1.5.

For the case of the DIV VSD, the WT-β3-EGFP and mutants had little effect on *V*_½_ for the S4 helix movement relative to Nav1.5 α-subunit alone. However, WT-β3-EGFP caused the initial activation of the DIV-VSD to occur at a more positive potential than in the absence of β3 and showed a greater voltage dependence of activation, with a reduced slope factor for the S4 helix movement in DIV VSD. These effects of β3 were not altered by the presence of the E176K mutation. The β3-ΔECD-EGFP single mutation activated the DIV-VSD at more negative potentials compared with WT-β3-EGFP. In addition, there was a significantly decreased voltage dependence of activation with increased slope *k* values compared with WT-β3-EGFP and E176K-EGFP, whereas the β3-ΔECD-E176K-EGFP double mutant produced a still greater increase in *k* (Fig. 6 and Table 3).

Taken together, these results suggest that the β3-subunit Glu-176 residue and the ECD both influence movement of the DIII VSD, albeit through distinct mechanisms. In addition, the ECD is also important in mediating the effects of the β3-subunit on the voltage dependence of the DIV VSD movement (Fig. 6 and Table 3), translating to the depolarizing shift of *V*_½_ inactivation exhibited with co-expression of full-length WT β3-subunit.

## Discussion

Previous work has shown that distinct structural regions and domains within the β3-subunit modify discrete aspects of Nav channel gating behavior (10, 15, 25). Here, we explore the functional significance of additional structural features within the β3-subunit protein: in particular, the Glu-176 residue within the transmembrane α-helix.

Glutamic acid is significantly under-represented within α-helical transmembrane sequences. This relative rarity probably reflects the thermodynamic cost of burying a potentially negatively charged side chain in a hydrophobic environment (18). In principle, such an unfavorable interaction could be compensated by allowing water molecules to surround the side chain or by an increase in the p*K_a_* of the glutamic acid side chain so that it remains largely protonated and uncharged when inside the membrane. However, both possibilities could have important structural consequences (17). Given these constraints, the striking evolutionary conservation of the Glu-176 residue strongly argues for its functional significance. Interestingly, the pattern of maximum sequence conservation throughout the entire transmembrane α-helix shows an approximate 4:3 periodicity. This will generate a helix where the maximally conserved residues, including Glu-176, all lie on the same face (Fig. 1, *B* and *C*). We therefore suggest that the Glu-176 residue is part of a larger feature, most likely a binding or interaction site.

The Nav channel β-subunits are members of the immunoglobulin superfamily and are related to members of the cell-adhesion N-CAM family. Indeed, Nav β-subunits can function as cell-adhesion molecules independently of the Nav α-subunits (26). Nav β-subunits can also adopt multiple oligomeric states in the absence of Nav α-subunits. For example, the β2 and β4-subunits can assemble as both *cis* and *trans* oligomers to promote *trans* cell adhesion (27). Similarly, the full-length β3-subunit can form *cis*-interacting dimers and trimers when expressed in cells without the Nav α-subunit (21). We have previously suggested that the membrane-embedded, protonated form of the Glu-176 residue could help stabilize these β3-subunit dimer and trimers by intersubunit hydrogen bonding (16, 21). However, our new data indicate that oligomerization of the full-length β3-subunit does not depend on the presence of the Glu-176 residue but depends on the presence of an intact ECD (Fig. 2*A*). This is consistent with structural data showing the purified ECD immunoglobulin domain can form trimers *in vitro* (21) and implies that the Glu-176 residue has been conserved for other purposes.

The location of the physiologically relevant β3-binding site on the Nav1.5 α-subunit is currently uncertain. Recent cryo-EM structures for the eel Nav channel, human Nav1.4, and human Nav1.7 have identified the binding site for the β1-subunit transmembrane region between the S1 and S2 helices of the DIII VSD (11, 28, 29). The transmembrane regions of the β1 and the β3-subunits share significant sequence similarity, and this includes a glutamic acid in the β1 sequence at the equivalent position to Glu-176 in β3 (9). It is therefore tempting to assume that the β1 and β3 transmembrane regions may bind to the same site, and previous VCF experiments do indeed indicate that the β3-subunit transmembrane region binds within the DIII VSD (13). However, several independent lines of evidence suggest that this hypothesis may be too simplistic. For example, in contrast to the results presented here, the equivalent Glu to Lys mutation in the transmembrane region β1 induced a significant shift in *V*_½_ for steady-state inactivation but had no effect on the recovery from inactivation (30). Then VCF experiments showed that unlike β3, the β1-subunit did not affect *V*_½_ or *k* for fluorescence changes in DIII for Nav1.5 (13). Furthermore, an introduced tryptophan in the β3-ECD neck connecting the Ig domain to the transmembrane region was able to quench the fluorescence signal from MTS-TAMRA selectively tagged at position 1296 of Nav1.5. This places the β3 neck region no more than 15 Å from residue 1296 of the Nav1.5 DIII, S3–S4 extracellular loop (13), a distance that is too short to be compatible with the known location of the β1-subunit transmembrane region on DIII between S1 and S2 (11, 12). Crucially, the same fluorescence quenching experiments, using an equivalent tryptophan mutation on the β1-subunit failed to detect quenching from Nav1.5 tagged with MTS-TAMRA at position 1296 (13). On this basis, we propose that the β3 transmembrane α-helix is more likely to lie between the Nav1.5 DIII S3 and S4 helices. This could allow the β3 ECD to interact with the DI-S5 and DIV-S5 extracellular loops but in a different orientation compared with β1 ECD. As a result, the full-length β3-subunit could potentially influence both DIII and DIV in a way that is distinct from β1.

As judged by immunoprecipitation, the β3-E176K-EGFP mutation interacted with the Nav1.5 α-subunit, although the amount of E176K β3 co-precipitated with Nav1.5 was somewhat reduced compared with WT β3 (Fig. 2*B*). This could reflect a somewhat lower affinity of the E176K β3 mutant for the Nav1.5 α-subunit, leading to lower retention of the E176K β3 mutant during the immunoprecipitation and extensive washing steps. However, both the E176K mutant and the WT β3-subunits induced a similar depolarizing shift in the *V*_½_ of Nav channel inactivation relative to that shown by the Nav1.5 α-subunit alone (Fig. 4 and Table 1). Therefore, the E176K mutant was still capable of functionally interacting with the Nav1.5 α-subunit *in vivo*.

To investigate the roles of the β3-subunit Glu-176 residue and its ECD, we used both patch-clamp electrophysiology and VCF. These two approaches provide complementary but different insights into Nav channel behavior. The electrophysiology reflects a culmination of all the steps and conformational changes that must occur before the channel can activate or inactivate. On the other hand, VCF experiments provide a more specific insight into voltage-dependent movements that occur in the S4 helices of individual VSDs, which subsequently lead to activation and/or inactivation (31).

The VCF experiments showed that in the absence of the β3-subunit, the *V*_½_ for the movement of the DIII S4 helix was nearly 50 mV more hyperpolarized than the equivalent value for the DIV S4 helix. Hence, for Nav1.5 alone, a depolarizing signal will initiate S4 helix movement considerably sooner in DIII than in DIV, and there will necessarily be a delay between the initiation of activation and subsequent inactivation pathways. Strikingly, however, the presence of full-length β3-subunit considerably reduced the difference between the *V*_½_ values for the DIII and DIV S4 helix movements (Fig. 6*B* and Table 3). By reducing this difference, the β3-subunit ensured that the DIII and DIV S4 helix movements occurred over a narrower voltage range and thus generated a more coordinated depolarization response from these two VSDs. Almost all the β3-induced relative changes in the *V*_½_ values occurred with the TAMRA-tagged C1296 Nav1.5 construct, suggesting that the DIII VSD is likely to interact directly with the β3-subunit.

It is striking that, compared with the WT-β3-EGFP, the full-length E176K mutant abrogated the effect of β3 on the voltage sensitivity of fluorescence changes for DIII VSD, while significantly increasing the slope factor *k* (Fig. 6*B* and Table 3). The slope factor, *k*, is inversely related to the amount of charge that can move across the membrane during a transition (32). Hence, the E176K mutant dramatically reduced this value, suggesting that without the β3-Glu-176 residue, the movement of the S4 helix in DIII during depolarization was compromised or at least altered relative to WT β3. A role for the Glu-176 residue in facilitating DIII S4 helix movement could also explain the effect of the β3-E176K-EGFP mutant on recovery from inactivation (Fig. 5 and Table 2). The DIII VSD is known to mediate recovery from inactivation *via* its interaction with the DIII-DIV linker region (14). By correctly positioning the DIII S4 helix in the inactivated state, the Glu-176 residue could in effect catalyze the β3-dependent accelerated recovery from inactivation as the membrane potential is repolarized.

Removal of the β3-subunit ECD (both with and without the E176K mutant) fully abrogated the effects of WT β3-subunit on the *V*_½_ for voltage-dependent fluorescence changes in DIII VSD (Fig. 6, *A* and *B*, and Table 3). This suggests that the ECD may play a role in correctly positioning the β3-subunit transmembrane α-helix. Interestingly, the co-immunoprecipitation experiments indicated that β3 constructs lacking the ECD bound to Nav1.5 more intensely compared with WT-β3-EGFP (Fig. 2*B*). It is therefore possible that the ECD may restrict the binding of the β3-subunit to one or a small number of physiologically appropriate sites on the Nav1.5 α-subunit. This could be achieved through the ECD Ig region interacting with specific extracellular loop regions on the α-subunit. Removal of the ECD could compromise this discrimination and enable the hydrophobic transmembrane region more freedom to bind additional sites on the α-subunit or to the same DIII region but with multiple orientations or conformational states, so that the Glu-176 residue is incorrectly positioned. In this case, the DIII S4 helix movement would no longer be so sensitive to the presence of the Glu-176 residue.

The DIV VSD plays an important role in steady-state inactivation (33). The WT-β3-EGFP subunit reduced the value of *k* for voltage-dependent S4 helix movements in DIV VSD (Fig. 6, *A* and *B*, and Table 3). This change was abolished for β3-subunits lacking the ECD (with or without the E176K mutation). Removal of the ECD also abolished the β3-induced depolarizing shift in steady-state inactivation gating (Fig. 4 and Table 1). Hence, the β3-ECD is likely to influence DIV, despite its location on the DIII VSD. One possibility is that the β3 ECD does this indirectly by modifying the conformation of the DI S5-S6 extracellular loop, adjacent to the DIV VSD. Alternatively, if the β3 transmembrane region binds to DIII VSD, it is likely that the ECD will interact with the DIV S5-S6 extracellular loop (Fig. S1), which could provide a more direct coupling between ECD and DIV. Furthermore, the magnitude of voltage-dependent fluorescence changes in DIII and DIV VSDs recorded with both β3-ΔECD-EGFP and β3-ΔECD-E176K-EGFP were less pronounced compared with Nav1.5 alone or with full-length β3 constructs (Fig. 6*A*). Because the fluorescence signal is very sensitive to changes in the immediate environment of the fluorophore (34), it is possible that removal of the ECD subtly altered the trajectory of the DIII and DIV S4 helix movements compared with Nav1.5 alone or the full-length β3 constructs.

In summary, our data provide new and quantitative insights into the unique role of the β3-subunit in the regulation of Nav1.5 gating. In this study, our emphasis has been to clarify the importance of distinctive and unusually positioned residues using the simplest and most tractable Nav1.5 α- and β3-subunits alone. In the future, building on this current work, it will be interesting to investigate these mutations in a more complex physiological system such as a cardiomyocyte, using, for example, targeted gene replacement methods.

## Experimental procedures

### Secondary structure predictions and modeling

Secondary structure prediction of the β3-subunit transmembrane region was carried out using Phyre2 (35). The alignment analysis of the β3 transmembrane region among different species was carried out using Clustal Omega program (36). A structural model of the β3-subunit was generated using the I-TASSER server (37).

### cDNA constructs, cell culture, mutagenesis, and cell transfections

Plasmids pcDNA3-Nav1.5-hemagglutinin (HA), pcDNA3 WT-β3-EGFP, and pcDNA3 β3-ΔECD-EGFP have been previously described (10, 15, 38). Site-directed mutagenesis of WT-β3-EGFP and β3-ΔECD-EGFP to introduce the E176K mutation was performed using the QuikChange Lightning kit (Agilent) with primers (designed complementary to the β3 sequence, except for the appropriate base change for glutamic acid to lysine (E176K) in the middle of the primer) from Sigma. Colonies were picked and grown in culture medium overnight, plasmids were then extracted using the Genelute plasmid midiprep kit (Sigma), and successful mutation was identified by Sanger sequencing (DNA sequencing facility, University of Cambridge). Separate Nav1.5 constructs containing the M1296C and S1618C were generated as previously described (24).

Human embryonic kidney (HEK293F) cells and HEK293F cells stably expressing Nav1.5 (HEK293F-Nav1.5) were maintained in Dulbecco's modified Eagle's medium (Dulbecco's modified Eagle's medium/F-12 GlutaMAX; Invitrogen) with 10% fetal bovine serum (Sigma–Aldrich) at 37 °C and 5% CO_2_. Transient transfections were performed using polyethylenimine (1 μg/μl) at a polyethylenimine/DNA ratio of 3:1. For whole-cell patch-clamp electrophysiology, HEK293F-Nav1.5 cells were plated on 18-mm coverslips in 6-well plates and transiently transfected with either 1 μg of the empty vector pEGFP-N1 or appropriate β3 plasmid DNA. For co-immunoprecipitation studies, HEK293F cells were co-transfected with 3 μg of Nav1.5 HA and 3 μg of either pEGFP-N1 or appropriate β3-GFP. For β3 cross-linking experiments, 8 μg of appropriate WT or mutant β3-GFP constructs were transfected.

### Co-immunoprecipitation

48 h post-transfection, the cells were washed three times in cold PBS and then lysed in 1 ml of lysis buffer (50 mm Tris, 150 mm NaCl, 1% Triton X-100 (v/v)) supplemented with protease inhibitor mixture (Roche, Sigma–Aldrich)). Lysates were vortexed, stored on ice for 30 min, and centrifuged at 10,000 × *g* for 10 min at 4 °C, and the pellet (cell debris) fraction was discarded. Lysates were incubated in either mouse anti-GFP tag or mouse anti-HA with end-over-end rotation at 4 °C overnight, followed by the addition of protein G–agarose for 4 h. The samples were spun at 2,000 × *g* at 4 °C for 5 min. Pellet (bound) fractions were washed four times in 1 ml of lysis buffer, and both these and the supernatants (unbound) were incubated in 4× NuPage loading buffer supplemented DTT at 85 °C for 10 min. The bound and unbound fractions were separated on SDS-PAGE and transferred to nitrocellulose membranes, and Western blotting was carried out with rabbit polyclonal antibodies anti-GFP (GeneTex, Insight Biotech) and anti-HA (Santa Cruz, Insight Biotech) to detect EGFP as well as the β3-GFP WT and mutants and Nav1.5-HA, respectively.

### BS3 cross-linking experiments

Transfected cells were washed three times in Hanks' balanced salt solution and then lysed in Hanks' balanced salt solution lysis buffer (1% Triton X-100, 0.2% SDS, 0.5% sodium deoxycholate) supplemented with protease inhibitors (Roche cOmplete Protease Inhibitor mixture). Each lysate was separated into two equal fractions, one of which was incubated with BS3 (5 mm) for 1 h at 4 °C. The reaction was quenched with glycine (74 mm), and the lysate was clarified at 10,000 × *g* for 10 min at 4 °C. The lysates were then subjected to SDS-PAGE and transferred to nitrocellulose membranes, and Western blotting was carried out with rabbit polyclonal antibodies anti-GFP (GeneTex, Insight Biotech) and anti-myc (Santa Cruz, Insight Biotech) to detect β3-GFP and β3-myc, respectively.

### Whole-cell patch clamp

Na^+^ currents (*I*_Na_) were recorded from HEK293F cells stably expressing Nav1.5 and transiently transfected with 1 μg of the β3 constructs or a vector containing only EGFP. Successfully transfected cells were identified by EGFP fluorescence on an Olympus IX71 inverted microscope. Experiments were carried out at room temperature (22–23 °C) in the whole-cell configuration with an Axopatch 200B amplifier (Axon Instruments), a Digidata 1322A digitizer (Axon Instruments), and the Strathclyde Electrophysiology Software Package (WinWCP, Department of Physiology and Pharmacology, University of Strathclyde). The extracellular bath solution contained 60 mm NaCl, 2 mm KCl, 1.5 mm CaCl_2_, 10 mm glucose, 1 mm MgCl_2_, 90 mm CsCl_2_, 10 mm HEPES, pH 7.39 ± 0.02 with NaOH. 1.5–2.5 mΩ patch pipettes were produced from borosilicate glass capillaries (Harvard Apparatus Ltd.) using a horizontal puller (P-87; Sutter Instruments) and filled with intracellular saline comprised of 35 mm NaCl, 105 mm CsF, 10 mm EGTA, 10 mm HEPES, pH 7.39 ± 0.02 with CsOH. Signals were low-pass Bessel filtered at a frequency of 5 kHz and sampled at 125 kHz. Series resistance compensation was performed to 75–80%, and leak currents were subtracted using a P/4 protocol. The liquid junction potential (2 mV) was not corrected for. Data from cells with a current amplitude larger than 8 nA or with a clear loss of voltage control as demonstrated by poor I/V relationships were removed.

### Voltage protocols

All voltage protocols used a holding voltage of −120 mV. The steady-state inactivation and activation protocol consisted of a 100-ms depolarizing pulse ranging from −140 mV to +35 mV, in 5-mV increments, followed by a fixed −40 mV depolarizing pulse of 50-ms duration. Currents elicited from the first pulse constitute activation data and those from the second depolarizing pulse provide inactivation data.

Current traces were normalized against the whole-cell capacitance (*C*_m_), and the *I*/*V* relationship was plotted from the peak current at each test voltage. The values of Na^+^ conductance (*G*_Na_), for families of traces at each test voltage, were determined from the equation,

(Eq. 1) $$G_{\text{Na}}=I_{\text{Na}}/\left(V-E_{\text{Na}}\right)$$

where *I*_Na_ is the Na^+^ current and *E*_Na_ is the Na^+^ reversal potential. Peak *G*_Na_ was plotted as a function of voltage to produce activation curves. *I*_Na_ was normalized to the maximum elicited current and plotted against the conditioning voltage to yield inactivation curves. Both curves were fitted to the following Boltzmann function,

(Eq. 2) G/Gmax⁡=1/(1+exp⁡((V−V12)/k))

where *G*/*G*_max_ is the normalized conductance or current, *V*_½_ is the voltage of half-maximal activation or inactivation, *k* is the slope factor, and *V* is the test voltage or conditioning voltage.

Recovery from inactivation was examined using a double-pulse P1 and P2 protocol that delivered two identical depolarizing pulses to −40 mV of 50-ms duration. The time interval between P1 and P2 was initially incremented by 1 ms up to 6 ms, followed by 2-ms increments to 20 ms, then 5-ms increments to 60 ms, followed by 10-ms increments to 120 ms, and finally 20-ms increments to 200 ms to ensure enough time was allowed for full recovery and to allow adequate capture of the fast components. Peak currents from P2 were normalized to those obtained in response to the conditioning P1 step and plotted against the time intervals. These plots were fitted with a double exponential function as follows,

(Eq. 3) $$y=A_{1}\exp\left(-t/\tau_{1}\right)-A_{2}\exp\left(-t/\tau_{2}\right)$$

where *t* is the time, and τ is the time constant of recovery from inactivation.

### VCF in oocytes

cRNAs for the human Na_V_ β3 (UniProtKB/Swiss-Prot under accession no. Q9NY72) and α-subunit Na_V_1.5 (accession no. Q14524.1) were produced from the pBSTA and pMAX vectors, respectively. All mutagenesis was accomplished using the QuikChange II site-directed mutagenesis kit (Agilent), with primers from Sigma–Aldrich. Multiple colonies were picked, and plasmids were isolated using the NucleoSpin plasmid miniprep kit (Macherey–Nagel). After samples were confirmed with sequencing (Genewiz), a single clone was selected for a Midiprep preparation (NucleoBond Xtra Midi; Macherey–Nagel). PBSTA and pMAX plasmid were linearized with the NotI and SpeI restriction enzymes, respectively, and purified with the NucleoSpin gel and PCR clean-up kit (Macherey–Nagel). Finally, capped mRNA was synthesized *in vitro* using the mMESSAGE mMACHINE t7 transcription Kit (Life Technologies), purified via phenol-chloroform extraction, and reconstituted to a concentration of ∼1 μg/μl.

The mRNAs for the human α-subunit Na_V_1.5 and β3-subunits were injected at a 3:1 molar ratio (50–56 ng per cell total) into *Xenopus* oocytes. The oocytes were then incubated at 18 °C in ND93 solution (93 mm NaCl, 5 mm KCl, 1.8 mm CaCl_2_, 1 mm MgCl_2_, 5 mm HEPES, 2.5 mm sodium pyruvate, and 1% penicillin-streptomycin, pH 7.4). 5–7 days after injection, VCF recordings were performed. Before recording, the oocytes were labeled with 10 μmol/liter MTS-TAMRA (Santa Cruz Biotechnology) in a depolarizing solution (110 mm KCl, 1.5 mm MgCl_2_, 0.8 mm CaCl_2_, 0.2 mm EDTA, and 10 mm HEPES, pH 7.1) for 30 min on ice. Fluorescence data were collected simultaneously with ionic current on a custom rig as described previously (13, 24). Each fluorescence trace is a mean of 7–10 fluorescence recordings of the same cell.

## Author contributions

S. C. S., W. Z., C. L. H. H., J. R. S., and A. P. J. conceptualization; S. C. S., W. Z., Z. F. H., S. S. H., and data curation; S. C. S., W. Z., Z. F. H., S. S. H., C. L. H. H., J. R. S., and A. P. J. formal analysis; S. C. S., W. Z., C. L. H. H., J. R. S., and A. P. J. supervision; C. L. H. H., J. R. S., and A. P. J. funding acquisition; S. C. S., W. Z., Z. F. H., S. S. H., validation; S. C. S., W. Z., Z. F. H., S. S. H., J. R. I., investigation; S. C. S., W. Z., J. R. I., C. L. H. H., J. R. S., and A. P. J. methodology; S. C. S., W. Z., C. L. H. H., J. R. S., and A. P. J. writing-original draft; S. C. S., C. L. H. H., J. R. S., and A. P. J. project administration; S. C. S., W. Z., C. L. H. H., J. R. S., and A. P. J. writing-review and editing.

## Supplementary Material

Supporting Information

## Footnotes

5 The abbreviations used are:

VSD: voltage-sensing domain
BrS: Brugada syndrome
ANOVA: analysis of variance
EGFP: enhanced green fluorescent protein
VCF: voltage-clamp fluorometry
ECD: extracellular domain
BS3: bis-sulfosuccinimidyl suberate
HA: hemagglutinin
MTS-TAMRA: methanethiosulfonate-carboxytetramethylrhodamine.
