# Supplementary material for: Gating control of the cardiac sodium channel Nav1.5 by its β3-subunit involves distinct roles for a transmembrane glutamic acid and the extracellular domain
Source: J Biol Chem. 2019 Oct 28;294(51):19752–63. doi: 10.1074/jbc.RA119.010283 (PMC6926464; doi:10.1074/jbc.RA119.010283)
Supplement: Supporting Information [file supp_294_51_19752__index.html]

Gating control of the cardiac sodium channel Nav1.5 by its β3-subunit involves distinct roles for a transmembrane glutamic acid and the extracellular domain — β3 structures influence Nav1.5 gating — Gating control of the cardiac sodium channel Nav1.5 by its β3-subunit involves distinct roles for a transmembrane glutamic acid and the extracellular domain — β3 structures influence Nav1.5 gating — Supporting Information 

# Gating control of the cardiac sodium channel Nav1.5 by its β3-subunit involves distinct roles for a transmembrane glutamic acid and the extracellular domain

## Supporting Information

- Supplementary Figure S1 - Model of the Nav1.5 channel alpha-subunit, showing domains DI-DIV and individual residues described in the text.
