# Supplementary material for: Gating control of the cardiac sodium channel Nav1.5 by its β3-subunit involves distinct roles for a transmembrane glutamic acid and the extracellular domain
Source: J Biol Chem. 2019 Oct 28;294(51):19752–63. doi: 10.1074/jbc.RA119.010283 (PMC6926464; doi:10.1074/jbc.RA119.010283)
Supplement: Supporting Information [file supp_RA119.010283_154374_2_supp_417644_pzvshx.pdf]

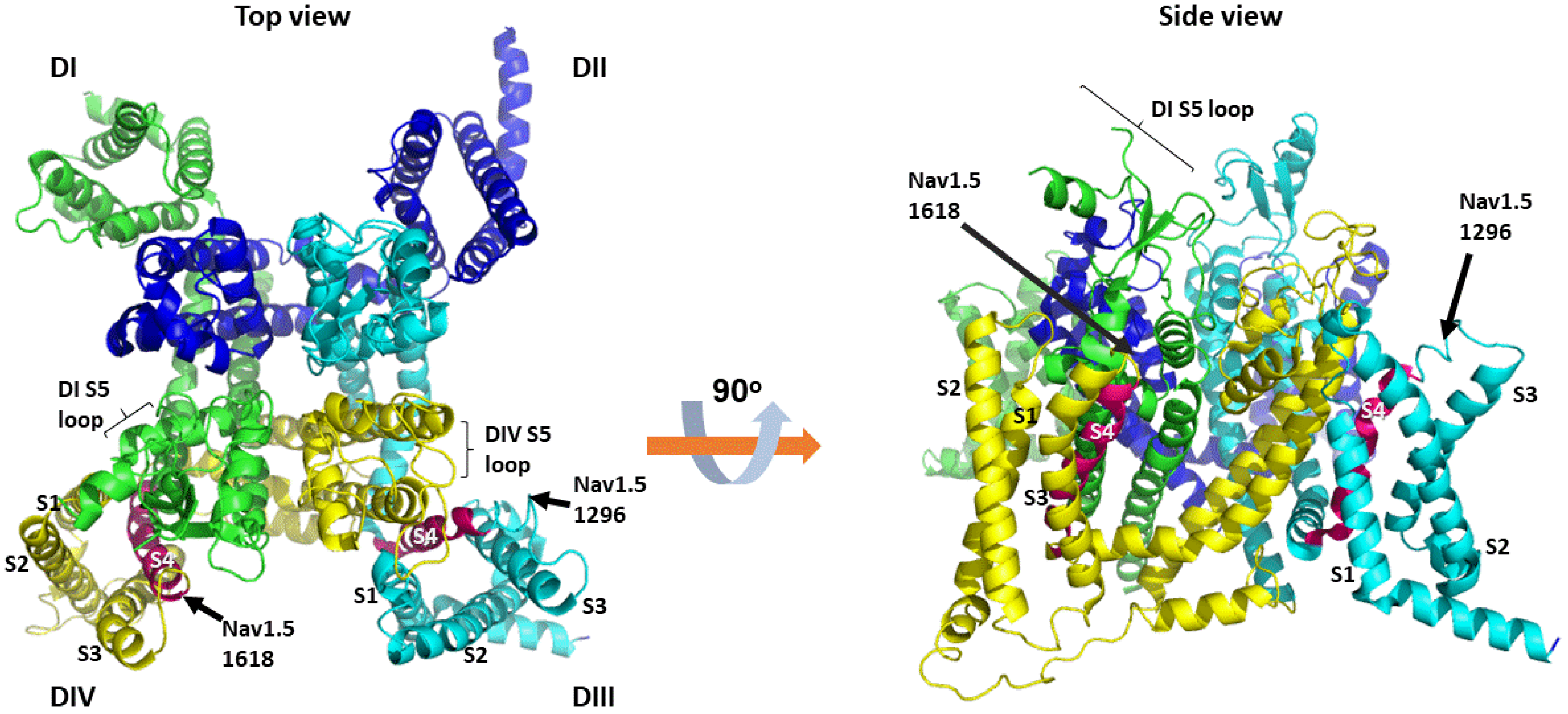

Supplementary Figure S1: Model of the Nav1.5 channel α-subunit, showing domains DI-DIV and individual residues described in the text. Each of the four internally homologous domains are differently colored. The VSDs of each domain comprise helices S1-S4 and are indicated for DIII and DIV VSD. The DI S5 and DIV S5 extracellular loop regions discussed in the text are indicated by brackets. The DIII and DIV S4 helices are colored purple for clarity. The location of residue 1296 in DIII VSD and 1618 in DIV VSD, that are selectively modified with MTS-TAMRA for the VCF experiments, are indicated by arrows. The model is based on the cryo-EM structure of human muscle Nav1.4 (PDB: 6AGF).
